# Distributed Sensor Network Calibration Under Sensor Nonlinearities with Applications in Aerodynamic Pressure Sensing

**DOI:** 10.3390/s25082505

**Published:** 2025-04-16

**Authors:** Srdjan S. Stanković, Miloš S. Stanković, Mladen Veinović, Ivana Jokić, Miloš Frantlović

**Affiliations:** 1 School of Electrical Engineering, University of Belgrade, 11020 Belgrade, Serbia; stankovic@etf.rs; 2 Faculty of Informatics and Computing, Singidunum University, 11010 Belgrade, Serbia; mveinovic@singidunum.ac.rs; 3 Vlatacom Institute, 11070 Belgrade, Serbia; 4 University of Belgrade—Institute of Chemistry, Technology and Metallurgy—National Institute of the Republic of Serbia, University of Belgrade, 11000 Belgrade, Serbia; ijokic@nanosys.ihtm.bg.ac.rs (I.J.); frant@nanosys.ihtm.bg.ac.rs (M.F.)

**Keywords:** aerodynamic pressure sensing, blind macro-calibration, bounded input–bounded output stability, dynamic consensus schemes, sensor networks, sensor nonlinearities

## Abstract

The theoretical part of this paper is devoted to a class of distributed blind calibration algorithms for large sensor networks based on consensus. The basic blind calibration method starts from affine sensor models and calibration functions, aiming to equalize corrected sensor offsets and gains without requiring any a priori knowledge of the measured signal. The main focus is to systematically and rigorously analyze the behavior of the calibration algorithms of the stochastic approximation type under nonlinear sensor models and stochastic environments, and to provide recommendations that are relevant to practice. It is demonstrated that the calibration algorithm—based on consensus with respect to all the calibration parameters—is far less robust to unknown sensor nonlinearities than the modified algorithm, taking one micro-calibrated sensor as a reference. Stability proofs of the algorithms are given in the bounded input–bounded output sense. The influences of measurement and communication noises are also analyzed using the theory of stochastic approximation. Numerous simulation results provide a comprehensive picture of the algorithm properties that are relevant to practice. This is followed by an important verification of the theoretical results, obtained by applying the analyzed blind calibration algorithms to an originally designed multichannel instrument for aerodynamic pressure sensing. A description of the new instrument is given, together with essential aspects of the implementation of the blind calibration algorithm. It is shown that the selected algorithm can be seen as a simple and efficient practical tool for blind online real-time re-calibration of complex sensor networks during normal system operations.

## 1. Introduction

In recent years, we have witnessed increasing interest in the diverse applications of large *sensor networks*, which are composed of a large number of sensors of different kinds. Sensor networks represent important parts of Cyber–Physical Systems (CPSs), the Internet of Things (IoT), large-scale systems, and networked control systems (see, e.g., [1,2,3,4,5,6,7,8]). In a majority of cases, sensor networks are used for *supervision and monitoring* purposes, e.g., the monitoring of air characteristics (the concentrations of different gases, particles, etc.), environment properties (temperature, humidity, and radioactivity), and wind properties; they can also be parts of feedback control for large-scale systems, systems of systems, etc. Important applications of sensor networks can also be found in research and development laboratories to support complex experiments.

The calibration of large sensor networks represents a great challenge from the point of view of *concept, technology, and organization*, bearing in mind the always-present need for high-precision measurement data in spite of potentially insufficient sensor quality and hostile environments. There are different formal definitions of the *sensor network calibration problem* [9,10,11,12,13]. Classical calibration procedures are usually performed in controlled environments; when this is not possible, or when sensors are deployed at barely accessible positions, calibration can be performed with respect to (1) pre-calibrated reference node(s), (2) selected sets of calibrated nodes (*distributed calibration*), and (3) non-calibrated nodes (*blind calibration*) [13]. Notice also that a large number of sensors, even those dedicated to important and sensitive functions, are often sold without *individual sensor calibration* (so-called *micro-calibration*, usually performed under strictly controlled conditions [13]). The calibration of a sensor network as a whole may be necessary for the successful deployment of large CPS and IoT systems, bearing in mind the inherent imperfections of the devices, the time variations of sensor parameters, the noise in acquired data, and random disturbances in the environment.

The development of calibration methodologies that are applicable at the level of a *sensor network as a whole* represents a great theoretical and practical challenge. According to some authors, *macro-calibration* deals with the calibration problem at the network level, e.g., [13,14,15,16]; meanwhile, macro-calibration, *without known stimuli*, is identified as the problem of *blind macro-calibration*, which is very complex and conceptually related to the problem of blind deconvolution [17,18,19,20,21]. The existing approaches to macro-calibration start from different a priori assumptions and use different methodologies, but they typically require centralized actions and non-recursive computations, e.g., [13,14,15,22,23,24]. Different macro-calibration methodologies can be found in the literature, including signal space projection [22,25], sparsity and convex optimization [26], consistency maximization [27], Kalman filtering [28], sparse Bayesian learning [29], deep learning [30], autoregressive signal modeling, matrix factorization [31], and consensus-based clustering [32] (additional examples can be found in a short survey within [33]).

It is actually even more challenging, both conceptually and practically, to develop efficient *decentralized algorithms for the blind macro-calibration* of sensor networks. One of the rare methodologically consistent solutions to this problem can be found in [34,35,36]. A comprehensive picture of the most important theoretical and practical aspects of the underlying ideas in this approach is presented in [33]. The proposed algorithms are of the *distributed-gradient type*, providing equal corrected sensor outputs by ensuring convergence to the *consensus* of *all the corrected sensor gains and offsets*, without requiring any type of function centralization. The main intuitive motivation for such an approach is the fact that identical readings of all sensors provide the following: (1) uniformly good measurement results in the case of the majority of high-quality sensors; (2) an important possibility of obtaining ideal calibration of the whole network by the preliminary micro-calibration of only one selected sensor—this is taken later as a reference (leader) in an algorithm, ensuring the equality of all the asymptotic corrected sensor gains and offsets to those introduced by the reference node. The mentioned basic algorithms have been modified in such a way as to provide even more flexibility, proposing the separate calibration of sensor gains using sensor output increments; this methodology has its roots in the distributed time-synchronization problem [37,38,39,40,41,42,43]. In the literature, we can find related studies based on an entirely deterministic setting [44], on a stochastic setting (including measurement and communication-noise- and gossip-based asynchronous communications between the sensors [45]), and on nonlinear calibration algorithms aiming to achieve robustification under the influence of outliers in the form of impulsive stochastic disturbances [46]. It is to be noted here that all the algorithms in both sets of papers are essentially based on *affine sensor models* and *affine calibration functions*.

In this paper, we are going to make a step further in the development of the calibration methodology from [34,35,36,44,45,46,47]. We provide a generalization of the existing results by considering *nonlinear sensor models*, aiming to investigate robustness with regard to the influence of unknown nonlinear inputs, representing the difference between the real sensor characteristics and the initially introduced affine model. We adopt a specific strategy for *gain calibration* [44,45,46,47], based on autonomous gain calibration due to its high flexibility in practice. We define the figure of merit of the following: (1) the basic algorithms applying full consensus, including all the network nodes; (2) algorithms obtained by fixing one node to an a priori defined reference value, in order to select algorithms possessing good stability properties and high robustness to noise and parameter changes. In this sense, we propose a number of originally formulated theorems providing the B.I.B.O. (bounded input–bounded output) stability of the algorithms and indicating their capabilities to reach consensus (Section 3, Section 4, Section 5 and Section 6). We illustrate the obtained theoretical results by simulation (Section 7) and apply the best algorithm to an ad hoc sensor network, designed and implemented in an originally developed multichannel aerodynamic pressure-sensing instrument (Section 8). To the authors’ knowledge, similar or comparable results cannot be found in the literature.

The first part of the paper (Section 2, Section 3, Section 4, Section 5 and Section 6) is dominantly theoretical, providing several original contributions concerning the stability properties of the algorithms. In Section 2, we briefly give a new definition of sensor outputs, containing a general static nonlinear term and stochastic disturbances in the form of additive measurement and communication noises. Section 3 is devoted to *offset calibration*. Within two theorems, we derive explicit formulae for the evolution of corrected offsets in the following cases: (1) the generic full-consensus-based calibration algorithm; (2) an algorithm based on pinning the algorithm to a selected reference node (sensor) in such a way as to fix the corrected offset as a reference value. It is proved that, in both cases, we can obtain the B.I.B.O. stability of the algorithms, with one substantial property: in the first case, the expected value at consensus is in the form of a weighted sum of the initial parameter estimates; in the second case, it is directly the predefined reference value (ideally equal to zero). In Section 4, we analyze algorithms for gain calibration based on the approach adopted in [45], using measurements of *sensor output increments*. A detailed analysis shows that even the existence of small nonlinear terms in the sensor models make the full-consensus-based algorithm inapplicable. The emphasis is placed on the algorithm derived by fixing the corrected sensor gain of one selected sensor, similarly as in the case of offset correction. It is proved in Theorem 3—using the arguments related to slowly varying linear dynamic systems—that the algorithm is B.I.B.O.-stable, and, therefore, applicable in practice. Section 5 is concerned with *simultaneous offset and gain calibration*. After deriving a general algorithm generating offset and gain corrections, we demonstrate that the algorithm with full consensus is again not reliable in practice, and that the algorithm with reference with regard to both offsets and gains ensures B.I.B.O. stability. In Section 6, attention is paid to the effects of the *stochastic environment*, encompassing *additive measurement and communication noises*. In the introductory part, we demonstrate how to extend the results related to the deterministic case (in Section 2, Section 3, Section 4 and Section 5). A concise presentation is given for the offset and gain-correction algorithms under additive measurement and communication noises, indicating their mean square convergence to consensus. Section 7 is devoted to carefully chosen *simulation examples* that illustrate the conclusions formulated in the preceding sections.

Section 8 is devoted to the following: (a) a description of a newly developed *multichannel instrument for aerodynamic pressure sensing*, dedicated to aerodynamic testing of fast-moving objects, or large structures under the influence of wind; (b) a description of an implementation of the above-presented distributed blind calibration algorithm embedded in the newly developed instruments; (c) the presentation of the results of blind calibration of the newly developed instrument during normal system operation. We first provide an introduction into aerodynamic pressure measurements, followed by a short description of a novel multichannel pressure-sensing instrument with improved properties with respect to the existing ones. Then, we define the calibration problem to be solved, and introduce the corresponding assumptions based on both the instrument’s design and the way the aerodynamic testing experiments are typically carried out. We point out that the developed calibration algorithms are well suited for online and real-time blind calibration of the developed instruments. After paying attention to the general aspects of the implementation of the proposed calibration algorithms, we present and discuss some initial results of calibration under normal system operation. These results represent an indication of how to calibrate networks of complex measurement instruments.

### Research Methodology

The research presented in this paper is of multidisciplinary character, encompassing the following attributes: (1) very sophisticated and complex calibration algorithms of the stochastic approximation type based on consensus; (2) the design and implementation of a new, original multichannel aerodynamic pressure-sensing instrument; (3) the incorporation of new original blind calibration algorithms in the software tools, supporting the normal functioning of the developed instrument. Summarizing the main lines of the adopted research methodology, we would like to better clarify the main contributions.

The general goal has been to develop a blind calibration method which could enable the online and real-time blind calibration of complex sensor networks; this would be a complementary tool with regard to the standard offline calibration methods, based on the generation of special test or reference signals.

The first step is of a theoretical nature, and provides the background for constructing a distributed and recursive calibration algorithm that will be applicable under the existence of static nonlinearities and additive noise. Adopting the distributed blind calibration methodology presented recently in a number of renowned journals [33,34,35,36,37,43,44,45,46,47], the presented theoretical results contain several new, rigorously proven theorems, providing evidence for the merit of the two main algorithms: (1) an algorithm based on full consensus; (2) an algorithm based on a reference node, usually micro-calibrated in advance. It has been proven that the second alternative is much more robust and is still simple enough from the points of view of both computations and communications. A number of illustrative figures are given to provide a general qualitative picture of the whole calibration methodology. Notice that the given results represent, to the authors’ knowledge, the first systematic analysis of the calibration algorithms proposed in [33,34,35,36,37,43,44,45,46,47] under the influence of sensor nonlinearities.

The second step follows after the design and implementation of a sophisticated MEMS multisensor instrument for measuring aerodynamic pressure, dedicated to complex experiments. In this sense, the fundamental conclusion about typical operating regimes shows that there exists a possibility of applying the theoretically analyzed calibration methods to a network of newly developed instruments and to expect an overall improvement in precision. This paper contains a presentation of the initial experimental results, but we hope that it opens up new perspectives. In such a way, the presented results enable us to draw the following conclusion: the developed methodology can be taken as a firm basis for the requirements that emerge in the complex experiments that are undertaken when using the developed instrument.

## 2. Problem Formulation

In this section, we present the general aspects of the distributed blind calibration problem by generalizing the approach based on affine sensor models presented in the previously published group of papers [34,35,36,44,45,46].

Consider *n*-distributed sensors to be measuring an *unknownsignal*, $x_{t}$, at discrete time instants, *t*, $t\in I^{+}$, where $I^{+}$ is a set of non-negative integers. The local *output* of the *i*-th sensor is defined by the following *general model*:

(1) $$y_{t}^{i}\left(x_{t}\right)=\beta^{i}+\alpha^{i}x_{t}+v^{i}\left(x_{t}\right)+\xi_{t}^{i},$$

where the *offset*, $\beta^{i}$, and the *gain (drift)*, $\alpha^{i}>0$, are *unknown* constants; $v^{i}\left(x_{t}\right)$ is an unknown bounded function, modeling the part of the sensor output that is not explained by the linear model, $\beta^{i}+\alpha^{i}x_{t}$; $\xi_{t}^{i}$ is the local *additive measurement noise*, i=1,…,n.

The *calibration* of each sensor is performed by applying the following *affine calibration transformation*, producing the *corrected sensor output*:

(2) $$z_{t}^{i}=b^{i}+a^{i}y_{t}^{i}=f^{i}+g^{i}x_{t}+a^{i}v^{i}\left(x_{t}\right)+a^{i}\xi_{t}^{i},$$

where $a^{i}$ and $b^{i}$ are the *gain (drift) correction* and the *offset correction* *(calibration)* *parameters*, respectively; $g^{i}=\alpha^{i}a^{i}$ in (2) represents the *corrected gain (drift)*; $f^{i}=a^{i}\beta^{i}+b^{i}$ represents the *corrected offset*.

**Remark** **1.** *A simple idea behind the calibration transformation (2) is to achieve $g^{i}\approx 1$ and $f^{i}\approx 0$ through a proper choice of the calibration parameters $a^{i}$, $b^{i}$, and i=1,…,n. The simplified case, corresponding to $v^{i}\left(x_{t}\right)=0$, ∀t, has been treated in the vast majority of papers dealing with sensor calibration, proposing different methodologies for determining $a^{i}$ and $b^{i}$ (see the Introduction and the references therein). However, the fact that the sensor parameters $\alpha^{i}$ and $\beta^{i}$ are unknown and the fact that there is no information about the measured signal, $x_{t}$, make the blind calibration problem highly nontrivial.*

**Remark** **2.** *In this paper, we assume that $x_{t}$ is an unknown bounded deterministic function of time, implying a deterministic character of $v^{i}\left(x_{t}\right)$. Therefore, the analysis presented below will be based on deterministic concepts and the stability properties of deterministic time-varying systems. Notice that an alternative approach is possible, based on the assumption that $x_{t}$ is a random sequence (see, e.g., [33,44,45,46]).*

We assume that the observed sensors form a *network* with specific structural properties, which can be formally represented by a *directed graph* (digraph), G(N,E), where N is the set of nodes and E is the set of directed edges (arcs) [33,44,48]. Let $A_{ad}=\left[a_{ad}^{ij}\right]$ be the n×n *adjacency matrix* of G, such that $a_{ad}^{ij}=1$ when the *j*-th sensor sends its messages to the *i*-sensor; otherwise, $a_{ad}^{ij}=0$. Let $N_{i}^{in}$ represent the set of the in-neighboring nodes of node *i*, i.e., the nodes satisfying $a_{ad}^{ij}=1$ for a given *i* and all *j*; $N_{i}^{out}$ represents the out-neighborhood of node *i*.

In the following, we start from the above sensor and calibration models and require that the corrected sensor outputs, $z_{t}^{i}$ (and/or their increments $\Delta z_{t}^{i}$), are *asymptotically equal*, without any information of the measured signal, $x_{t}$. A simple interpretation of this approach in practice is to exploit the dominant influence of the well-calibrated sensors, which are in the majority in typical exploitation conditions. Below, we give an exact formal definition of the underlying criteria and demonstrate that adequate a priori sensor weighting is quantitatively ensured by a set of meta-parameters strongly influencing the character of the result (see (3) and (15) below). We construct recursive estimators of the calibration parameters $b^{i}$ and $a^{i}$ by assuming that the sensor outputs, $y_{t}^{i}$, are locally available and the nodes interchange messages according to a given adjacency matrix, $A_{ad}$, in such a way that each message between the selected nodes, *j* and *i*, is corrupted by *additive communication noise*, $\eta_{t}^{ij}$.

**Remark** **3.** *It is to be noted at this point that all the sensors measure the same signal, $x_{t}$ (observed through sensor outputs $y_{t}^{i}$). In large sensor networks spreading over large 2D or 3D spaces, this assumption may not be justified. Then, a straightforward approach would be to build a spacial model, connecting signals at different spacial coordinates according to spacial correlation functions, and to incorporate such a model in the calibration procedure (see, e.g., [23]). Notice that, in some cases, there exist special working regimes of sensor network environments within large networked control systems, which justify the assumption that all sensors observe the same signal; one practically important example is described in Section 8 in relation to complex experiments dealing with aerodynamic pressure sensing.*

## 3. Offset Correction

In this section, we present two distributed recursive calibration algorithms for estimation of the calibration parameters, $b^{i}$, in (2), assuming that the sensor gains are ideal, i.e., $\alpha^{i}=1$. The algorithm for isolated offset calibration has been formulated in [44]; it represents a special case of the calibration algorithms presented in [33,34,44]. Isolated offset estimation can often be encountered in practice, since the sensor offset is usually the most sensitive parameter requiring re-calibration. We also present the basic mathematical tools used throughout the paper.

### 3.1. Full-Consensus Algorithm

Consider a special case of the model (1) and the calibration function (2) in which $\alpha_{i}=1$, $\xi_{t}^{i}=0$, and $x_{t}$ is an unknown deterministic function of *t*, bounded for all *t*. According to the general consensus-based calibration methodology presented in [34,35,36,44,45,46], we define the following criterion function:

(3) $$J_{b}^{i}\left(b^{i}\right)=\sum_{j\in N_{i}^{in}}\gamma_{ij}{\left(z_{t}^{j}-z_{t}^{i}\right)}^{2},$$

i=1,…,n, where $\gamma_{ij}$ are *non-negative weights* reflecting the relative importance of neighboring nodes from $N_{i}$ for all *i*. After computing the derivatives of $z^{i}$ with regard to $b^{i}$, we can directly formulate the following distributed *gradient-type algorithm* for estimating $b^{i}$ in real time [49,50,51], using the current measurements of the sensor outputs:

(4) $${\hat{b}}_{t+1}^{i}={\hat{b}}_{t}^{i}+\delta_{t}^{i}\sum_{j\in N_{i}^{in}}\gamma_{ij}\varepsilon_{t}^{ij},$$

where ${\hat{b}}_{t}^{i}$ is an estimate of $b^{i}$ at time *t*, $\delta_{t}^{i}$ is a positive step-size sequence influencing the convergence properties of the algorithm, and $\varepsilon_{t}^{ij}={\hat{z}}_{t}^{j}-{\hat{z}}_{t}^{i}$, with ${\hat{z}}_{t}^{i}=y_{t}^{i}+{\hat{b}}_{t}^{i}$, where $y_{t}^{i}$ is defined by (1). The initial conditions are determined by the available a priori information; without any information, it is natural to adopt ${\hat{b}}_{0}^{i}=0$, i=1,…,n. Notice that, to the authors’ knowledge, the nonlinear term $v_{t}^{i}=v^{i}\left(x_{t}\right)$ has not been treated in this context in the literature.

It is convenient to reformulate (4) in the following way:

(5) $${\hat{f}}_{t+1}^{i}={\hat{f}}_{t}^{i}+\delta_{t}^{i}\sum_{j\in N_{i}^{in}}\gamma_{ij}\left\{\left({\hat{f}}_{t}^{j}-{\hat{f}}_{t}^{i}\right)+\left(v_{t}^{j}-v_{t}^{i}\right)\right\},$$

where ${\hat{f}}_{t}^{i}$ is an estimate of the corrected offset $f^{i}$. All the recursions (5) can be represented by the following global vector–matrix model at the network level:

(6) $${\hat{f}}_{t+1}=\left(I+\Delta_{t}\Gamma \right){\hat{f}}_{t}+\Delta_{t}\Gamma v_{t}$$

where ${\hat{f}}_{t}={\left[{\hat{f}}_{t}^{1}\cdots {\hat{f}}_{t}^{n}\right]}^{T}$, $\Delta_{t}=\mathrm{diag}\left\{\delta_{t}^{1},\ldots ,\delta_{t}^{n}\right\}$,

$$\Gamma =\left[\begin{array}{cccc} -\sum_{j=2}^{n}\gamma_{1j} & \gamma_{12} & \cdots & \gamma_{1n}\\ \gamma_{21} & -\sum_{j=1,j\neq 2}^{n}\gamma_{2j} & \cdots & \gamma_{2n}\\ \vdots & \vdots & \vdots & \vdots \\ \gamma_{n1} & \gamma_{n2} & \cdots & -\sum_{j=1,j\neq n}^{n}\gamma_{nj} \end{array}\right]$$

and $v_{t}={\left[v_{t}^{1}\cdots v_{t}^{n}\right]}^{T}$.

The recursion (6) represents a linear dynamic system with time-varying parameters and unknown inputs, which can be analyzed using the general theory of linear time-varying systems, together with the specific methodology developed for dynamic consensus schemes, e.g., [52,53,54]. We adopt the following basic assumptions:

A1: $\delta_{t}^{i}=\delta =\mathrm{const}$.
A2: $∥v_{t}^{i}∥\leq k_{v}^{i}<\infty$, i=1,…,n.
A3: Graph G possesses a central node.

Assumption A1 is adequate for the adopted deterministic framework. In practice, it is advisable to adopt A1 when the tracking of time-varying parameters may be desirable. Assumption A3 is frequently encountered in consensus-based schemes (the central node is a node which can be reached from all the remaining nodes in the graph, G) [48].

The following two lemmas are fundamental for all the formal results presented in this paper. Matrix Γ, containing the algorithm meta-parameters, plays a fundamental role in defining the properties of the algorithm. A more detailed insight into the influence of Γ can be found in [44,45].

**Lemma** **1** ([55])**.** *Let A3 be satisfied. Then, matrix* Γ *has one eigenvalue at the origin and the remaining ones in the left-half complex plane.*

Define $1={\left[1\cdots 1\right]}^{T}$; vector 1 is, by definition, the right eigenvector of Γ, corresponding to the zero eigenvalue. Let π be the left eigenvector of Γ, corresponding to the zero eigenvalue, and satisfying πΓ=0 and π1=1.

**Lemma** **2** ([55,56,57])**.** Let $T=[1\vdots T^{*}]$, where n×(n−1) matrix $T^{*}$ satisfies $\mathrm{span}T^{*}=\mathrm{span}\left\{\Gamma \right\}$. Then, $T^{-1}=\left[\begin{array}{c} \pi \\ S^{*} \end{array}\right]$, where $S^{*}$ is an (n−1)×n matrix satisfying $S^{*}T^{*}=I_{n-1}$ and

$$T^{-1}\Gamma T=\left[\begin{array}{cc} 0 & 0_{1\times (n-1)}\\ 0_{(n-1)\times 1} & \Gamma^{*} \end{array}\right],$$

where $\Gamma^{*}$ is Hurwitz.

**Theorem** **1.** *Let A1–A3 be satisfied. Then, there exists $\delta^{\prime }>0$, such that, for all $0<\delta <\delta^{\prime }$, we have for all t the following solution of (6):*

(7) $${\hat{f}}_{t}=\pi {\hat{f}}_{0}1+S^{*}{\tilde{f}}_{t}^{\left[2\right]},$$

*where ${\tilde{f}}_{t}^{\left[2\right]}$ is generated by the following recursion:*

(8) $${\tilde{f}}_{t+1}^{\left[2\right]}=\left(I+\delta \Gamma^{*}\right){\tilde{f}}_{t}^{\left[2\right]}+\delta \Gamma^{*}{\tilde{v}}_{t}^{\left[2\right]}$$

with ${\tilde{f}}_{0}^{\left[2\right]}=S^{*}{\left[\beta^{1}\cdots \beta^{n}\right]}^{T}$, ${\tilde{v}}_{t}=T^{-1}v_{t}=$$[{\tilde{v}}_{t}^{[1]T}\vdots$${\tilde{v}}_{t}^{[2]T}{]}^{T}$, $\dim\{{\tilde{v}}_{t}^{\left[1\right]}\}=1$, $\dim\{{\tilde{v}}_{t}^{\left[2\right]}\}=n-1$. Furthermore, $∥\;{\tilde{f}}_{t}^{\left[2\right]}∥\;<\;\infty$ and $\lim∥v_{t}∥\;\to \;0$ implies $∥\;{\tilde{f}}_{t}^{\left[2\right]}∥\;\to \;0$.

**Proof.**  Introduce ${\tilde{f}}_{t}=T^{-1}{\hat{f}}_{t}$  [33,34,44,55]; then, if ${\tilde{f}}_{t}=$${\left[{\tilde{f}}_{t}^{[1]T}\vdots \;{\tilde{f}}_{t}^{[2]T}\right]}^{T}$, $\dim\{{\tilde{f}}_{t}^{\left[1\right]}\}=1$, $\dim\{{\tilde{f}}_{t}^{\left[2\right]}\}=n-1$, then (6) provides

(9) $${\tilde{f}}_{t+1}^{\left[1\right]}={\tilde{f}}_{t}^{\left[1\right]},$$

together with the recursion (8) for ${\tilde{f}}_{t}^{\left[2\right]}$, with ${\tilde{f}}_{0}=T^{-1}{\left[\beta^{1}\cdots \beta^{n}\right]}^{T}$. According to Lemma 2, $\Gamma^{*}$ is time-invariant and Hurwitz [52,55]; it follows that, for a sufficiently small $\delta^{\prime }$, the condition $\max_{i}\left|\lambda_{i}\left\{I+\delta \Gamma^{*}\right\}\right|<1$ holds. Therefore, (8) is exponentially asymptotically stable and also B.I.B.O.-stable [52], directly implying the assertion of the theorem. □

### 3.2. Algorithm with Reference

In the case when one of the sensors (say, w.l.o.g., the first) has been preliminarily micro-calibrated, its influence on the rest of the sensors in a network during calibration can be formally maximized in such a way as to adopt $\gamma_{kj}=0$, ∀j. The selected sensor can be then considered to be the *leader* of the whole sensor population (i.e., the network is considered to be *pinned* to the selected sensor), providing a *reference* for the whole population of sensors [33,34,44,55].

**Theorem** **2.** *Let A1–A3 be satisfied; then, suppose that a fixed reference value $f_{ref}^{1}$ is selected for the first sensor and that the node* 1 *is a center node of graph G. Then, there exists such a $\delta^{\prime \prime }>0$ that $\forall \delta <\delta^{\prime \prime }$; the algorithm (4), modified in such a way that it consists of*

(10) $${\hat{b}}_{t+1}^{1}={\hat{b}}_{t}^{1},$$

*with $b_{0}^{1}=-\beta^{1}$, and*

(11) $$r_{t+1}=\left(I+\delta \Gamma^{-}\right)r_{t}+\delta \Gamma^{-}v_{t}^{-},$$

*where $r_{t}={\left[r_{t}^{2}\cdots r_{t}^{n}\right]}^{T}$, $r_{t}^{i}={\hat{f}}_{t}^{i}-f_{ref}^{1}=$$\beta^{i}+{\hat{b}}_{t}^{i}-$$\left(\beta^{1}+{\hat{b}}_{t}^{1}\right)$, $v_{t}^{-}={\left[v_{t}^{2}\cdots v_{t}^{n}\right]}^{T}$,*

$$\Gamma^{-}=\left[\begin{array}{cccc} -\sum_{j=1,j\neq 2}^{n}\gamma_{2j} & \gamma_{23} & \cdots & \gamma_{2n}\\ \gamma_{32} & -\sum_{j=1,j\neq 3}^{n}\gamma_{3j} & \cdots & \gamma_{3n}\\ \vdots & \vdots & \vdots & \vdots \\ \gamma_{n2} & \gamma_{n3} & \cdots & -\sum_{j=1,j\neq n}\gamma_{nj} \end{array}\right],$$

*satisfies $∥r_{t}∥\;<\;\infty$ and $∥r_{t}∥\;\to \;0$ when t → ∞ and $∥v_{t}∥\;\to \;0$.*

**Proof.**  According to the general setting, we have the following n−1 local recursions:

(12) $${\hat{f}}_{t+1}^{i}={\hat{f}}_{t}^{i}+\delta \sum_{j=1,j\neq i}^{n}\gamma_{ij}\left[\left({\hat{f}}_{t}^{j}-{\hat{f}}_{t}^{j}\right)+\left(v_{t}^{j}-v_{t}^{i}\right)\right]$$

i=1,…,n, i≠1, bearing in mind that $r_{t}^{i}$ is defined by ${\hat{f}}_{t}^{i}$ and $f_{ref}^{1}$. Namely, the global recursion (11) is derived directly from (4) by putting all the underlying recursions together after fixing k=1 and ${\hat{b}}_{0}^{1}=-\beta^{1}$, in accordance with the assumption that the reference node has been a priori ideally micro-calibrated. Therefore, $f_{ref}^{1}=0$. In such a way, it follows that the recursion (11) is a time-invariant dynamic system with an input term depending on $v_{t}$, which is asymptotically exponentially stable for $v_{t}=0$, bearing in mind that $\Gamma^{-}$ is constant and Hurwitz, under the adopted assumptions (see, e.g., [34,44,45,52,53,58,59]). It is also B.I.B.O.-stable (for $v_{t}\neq 0$). The rest of the proof follows directly from [52] and Theorem 1. □

**Remark** **4.** *It is essential for the whole analysis to compare the results provided by (4) and (11) at a certain level of generality, in spite of the fact that the results are dependent on the parameters in Γ and the local sensor characteristics. It is obvious that there is a basic similarity between these algorithms. Both of them provide equal values of corrected offsets when $v_{t}=0$. However, in the case of (4), the common value of all the corrected offsets is given by $\pi {\hat{f}}_{0}$; meanwhile, in the case of (11), the common value is equal to zero for an ideally micro-calibrated sensor with $f_{ref}^{1}=0$. In general, it is natural that an additional local micro-calibration should enable us to obtain an initially better-corrected offset estimate. One should bear in mind that a new network quality can be obtained by acquiring the knowledge of one selected sensor output; in large sensor networks, this is not too demanding a requirement.*

**Remark** **5.** *Considering the basic term, $\varepsilon_{t}^{ij}={\hat{z}}_{t}^{j}-{\hat{z}}_{t}^{i}$, in (4), we can easily note that, in the case of a homogeneous population of sensors—in the sense that $y^{j}\left(x_{t}\right)\approx y^{i}\left(x_{t}\right)$ for all $x_{t}$—we have that $\varepsilon_{t}^{ij}\approx \beta^{j}-\beta^{i}-\left({\hat{b}}_{t}^{j}-{\hat{b}}_{t}^{i}\right)$, i.e., we can entirely rely on the linear sensor model and explicitly follow the conclusions from  [34,36,44,45,46]. This is an important property for practice, which can be utilized within the design of the pressure-measuring network presented later in Section 8.*

## 4. Gain Correction

Consider again the sensor network defined above, but assume that, in the general sensor model (1), the nonlinear term $v_{t}^{i}$ exists, the coefficients $\alpha^{i}$ are possibly different from each other and unknown, and the measurement noise term $\xi_{t}^{i}$ is absent, i=1,…,n. In this case, it is possible to follow either the methodology from [34,35,36] based on the joint estimation of offset and gain parameters or the methodology from [44,45], where the gain correction represents an autonomous process, based on the acquisition and processing of *measurement increments*. We adopt the latter approach, bearing in mind that it allows more flexibility for practice, i.e., the offset and gain parameters can be independently corrected.

### 4.1. Full-Consensus Algorithm

Starting from the approach in [44], we define $\Delta y_{t}^{i}=y_{t+1}^{i}-y_{t}^{i}$ and $\Delta x_{t}=x_{t+1}-x_{t}$; after replacing in (1), we obtain the following expression for the sensor output increments:

(13) $$\Delta y_{t}^{i}=\alpha^{i}\Delta x_{t}+\Delta v^{i}\left(x_{t}\right),$$

where $\Delta v^{i}\left(x_{t}\right)=v^{i}\left(x_{t+1}\right)-v^{i}\left(x_{t}\right)$. Let the calibration function be again given by (2).

The calibration goal is to find such calibration parameters $a^{i}$ which ensure asymptotical equality of the corrected gains $g^{i}=\alpha^{i}a^{i}$, i=1,…,n, as in the case of offset calibration. Obviously, we now have

(14) $$\Delta z^{i}=z_{t+1}^{i}-z_{t}^{i}=\alpha^{i}a^{i}\Delta x_{t}+a^{i}\Delta v^{i}\left(x_{t}\right).$$

We start from the following local criterion functions, formally expressing the basic calibration goal, i.e., the minimal squared difference between the sensor output increments:

(15) $$J_{a}^{i}\left(a^{i}\right)=\sum_{j\in N_{i}^{in}}\gamma_{ij}{\left(\Delta z_{t}^{j}-\Delta z_{t}^{i}\right)}^{2}.$$

The criterion (15) generates the following *distributed-gradient-type recursions* for estimating $a^{i}$, i=1,…,n:

(16) $${\hat{a}}_{t+1}^{i}={\hat{a}}_{t}^{i}+\delta_{t}^{i}\sum_{j\in N_{i}^{in}}\gamma_{ij}\varepsilon_{t}^{\Delta ,ij}\Delta y_{t}^{i},$$

where ${\hat{a}}_{t}^{i}$ is an estimate of $a^{i}$; meanwhile, $\varepsilon_{t}^{\Delta ,ij}=\Delta {\hat{z}}^{j}-\Delta {\hat{z}}^{i}$, with $\Delta {\hat{z}}^{i}={\hat{a}}_{t}^{i}\Delta y_{t}^{i}$ [49]. Initial conditions can be chosen arbitrarily; it is natural to adopt $a_{0}^{i}=1$ [44].

As in the case of offset correction, it is possible to obtain the following recursions of the corrected gains, ${\hat{g}}^{i}=\alpha^{i}{\hat{a}}_{t}^{i}$:

(17) $${\hat{g}}_{t+1}^{i}={\hat{g}}_{t}^{i}+\delta_{t}^{i}\Delta x_{t}^{2}\sum_{j}\gamma_{ij}\alpha^{i2}[{\hat{g}}_{t}^{j}\left(1+w_{t}^{j}\right)-{\hat{g}}_{t}^{i}\left(\left(1+w_{t}^{i}\right)\right]\left(1+w_{t}^{i}\right),$$

where we define $w_{t}^{i}=\frac{\Delta v_{t}^{i}\left(x_{t}\right)}{\alpha^{i}\Delta x_{t}}$. The following global model can be readily derived from (17):

(18) $${\hat{g}}_{t+1}={\hat{g}}_{t}+\delta \Delta x_{t}^{2}\alpha^{2}\Gamma_{t}^{w}{\hat{g}}_{t}$$

where

$$\Gamma_{t}^{w}=\Gamma +\Gamma W_{t}+W_{t}\Gamma +W_{t}\Gamma W_{t},$$

$g_{t}={\left[g_{t}^{1}\cdots g_{t}^{n}\right]}^{T},\;$α=$\mathrm{diag}\{\alpha^{1},\ldots ,\alpha^{n}\}$, $W_{t}=$$\mathrm{diag}\{w_{t}^{1},$$\ldots ,w_{t}^{n}\}$ (it is adopted, as above, that $\delta^{i}=\delta >0$).

**Remark** **6.** *Due to the fact that the corrected gain is estimated by using the increments of the measurement signal, the quantity $w_{t}^{i}$ represents the one-step increment (in time) of the nonlinear term in the sensor output, normalized by the increment of the measured signal itself multiplied by the gain from the sensor model.*

**Remark** **7.** *Notice that only the recursions (16) and (4) are implemented in practice in relation with the calibration parameters $a^{i}$ and $b^{i}$. The recursions for the corrected parameters $g^{i}$ and $f^{i}$, depending on the signals and parameters not available in practice, serve only as a tool for theoretical analysis.*

Obviously, the above gain-correction algorithm (17) does not depend on the sensor offsets, and can be applied independently [44,45]. However, its convergence remains an open question under the influence of sensor nonlinearities, keeping the structure of the global model in mind (18).

**Remark** **8.** *Recall that, in the case of the nonexistence of sensor nonlinearities (when $w_{t}=0$), the calibration algorithm converges exponentially to consensus under nonrestrictive conditions on the signal, $x_{t}$, itself [45]. Namely, we have in this case the following model:*

(19) $${\hat{g}}_{t+1}={\hat{g}}_{t}+\delta \Delta x_{t}^{2}\alpha^{2}\Gamma {\hat{g}}_{t}$$

*and we can prove—using the theory of time-varying linear stochastic systems and the theory of dynamic consensus schemes—that there exists such a $\delta^{\prime \prime }>0$ that, for all $\delta <\delta^{\prime \prime }$, the system asymptotically exponentially achieves consensus with regard to $g_{t}$ in the mean square sense [44,60].*

In order to obtain a qualitative insight into the convergence properties of (17) under the presence of sensor nonlinearities, we apply at this point the line of reasoning based on Theorem 1, and introduce ${\tilde{g}}_{t}={\tilde{T}}^{-1}{\hat{g}}_{t}$, where $\tilde{T}=\left[1\vdots {\tilde{T}}^{*}\right]$; here, the n×(n−1) matrix ${\tilde{T}}^{*}$ satisfies $\mathrm{span}\left\{{\tilde{T}}^{*}\right\}=\mathrm{span}\left\{\alpha^{2}\Gamma \right\}$. We obtain from (17) (keeping the definition of the matrix $\Gamma_{t}^{w}$ in mind) the following characteristic terms in the resulting expression for ${\tilde{g}}_{t}$:

(a) $\left({\tilde{T}}^{-1}\alpha^{2}\Gamma \tilde{T}\right){\tilde{T}}^{-1}{\hat{g}}_{t}$,
(b) $\left({\tilde{T}}^{-1}\alpha^{2}\Gamma \tilde{T}\right){\tilde{T}}^{-1}W_{t}{\hat{g}}_{t},$
(c) $\left({\tilde{T}}^{-1}\alpha^{2}W_{t}\Gamma \tilde{T}\right){\tilde{T}}^{-1}{\hat{g}}_{t},$
(d) $\left({\tilde{T}}^{-1}\alpha^{2}W_{t}\Gamma W_{t}\tilde{T}\right){\tilde{T}}^{-1}{\hat{g}}_{t}$.

It is straightforward to verify that, in cases (a) and (b), the vectors resulting from the given expressions have zeros as their first components (according to Lemmas 1 and 2); meanwhile, in cases (c) and (d), the first elements of these vectors are, in general, nonzero, depending on $W_{t}$. The first component of the transformed corrected parameter vectors is very important, since it is directly connected to the zero eigenvalue of $\alpha^{2}\Gamma$ [33,35,36,55]. Consequently, a comparison with Theorem 1 and the results from [34,44,52,55] shows that the desired consensus cannot be reached, in general, with regard to all the elements of ${\hat{g}}_{t}$, and that—even for small nonlinear terms, $v_{t}$—the whole system of recursions can become unstable. Observe that the existence of the time-varying terms $x_{t}$ and $v_{t}$ makes a reduction in ${\tilde{g}}_{t}^{i}$ to a single constant multiplied by 1 impossible; this is required for achieving consensus (compare with (9)).

**Remark** **9.** *Note that the formal elimination of the nonlinear term in the sensor model leads to an algorithm for which convergence has been proved in [44]. The assumption $v_{t}^{i}=0$ formally corresponds to the above term denoted as (a), when the consensus of the corrected gains is reachable.*

**Remark** **10.** *Similarly to Remark 5, when $\Delta y_{t}^{j}\approx \Delta y_{t}^{i}$ for all $x_{t}$, we have $\varepsilon_{t}^{\Delta ,ij}\approx {\hat{a}}^{j}\Delta x_{t}-{\hat{a}}^{i}\Delta x_{t}$, and we can again rely on the linear sensor model  [34,36,44,45,46].*

### 4.2. Algorithm with Reference

The above-identified problems with the basic algorithm for ${\hat{g}}_{t}$ given in (17) can be practically circumvented by introducing a *reference node*, similar to the case of offset calibration presented in Section 3.2. We assume again that the first node has been micro-calibrated in advance, so that $g_{ref}^{1}=\alpha^{1}{\hat{a}}_{t}^{1}=\alpha^{1}{\hat{a}}_{0}^{1}$ represents a reference gain (one can fix, in the ideal case, that ${\hat{a}}_{0}^{1}=1/\alpha^{1}$ after micro-calibrating sensor 1 in a convenient way). In practice, our desire is to construct an algorithm that is able to achieve that all ${\hat{g}}_{t}^{i}$, and i=2,…,n are asymptotically as close as possible to each other, at the same time ensuring a small distance from the ideal reference value.

Formally, we derive local recursions for $s_{t}^{i}$$={\hat{g}}_{t}^{i}-g_{ref}^{1}$ in a similar way as those for $r_{t}^{i}$ in the case of offset calibration, i=2,…,n. After straightforward manipulations with (19), we obtain

(20) $$s_{t+1}^{i}=s_{t}^{i}+\delta \Delta x_{t}^{2}\alpha^{i2}\sum_{j}\gamma_{ij}\left[\left(s_{t}^{j}-s_{t}^{i}\right)+\left(w_{t}^{j}{\hat{g}}_{t}^{j}-w_{t}^{i}{\hat{g}}_{t}^{i}\right)\right]\left(1-w_{t}^{i}\right).$$

Further, we have $\sum_{j=1}^{n}\gamma_{ij}w_{t}^{j}{\hat{g}}_{t}^{j}=\sum_{j=1}^{n}\gamma_{ij}w_{t}^{j}\left(s_{t}^{j}+1\right)$ (where we have included $g_{ref}^{1}=1$). Therefore, the algorithm model at the network level becomes

(21) $$s_{t+1}=s_{t}+\delta \Delta x_{t}^{2}{\left(\alpha^{-}\right)}^{2}\left[{\left(\Gamma_{t}^{w}\right)}^{-}s_{t}+\left(I+W_{t}^{-}\right)\Gamma^{-}w_{t}^{-}\right],$$

where $s={\left[s^{2}\cdots s^{n}\right]}^{T}$, $\alpha^{-}=\mathrm{diag}\left\{\alpha^{2},\ldots ,\alpha^{n}\right\}$, ${\left(\Gamma_{t}^{w}\right)}^{-}$ is obtained from matrix $\Gamma_{t}^{w}$ by extracting the sub-matrix corresponding to the indices i,j=2,…,n, and $w_{t}^{-}={\left[w_{t}^{2}\cdots w_{t}^{n}\right]}^{T}$. The global model (21) is a linear time-varying system, to which the B.I.B.O. stability result given below in Theorem 3 can be applied. It is to be noticed that additional time variations, making the analysis of (21) more complex than the analysis of (11), result from the term $\Delta x_{t}^{2}$. In order to apply the general theory of time-varying systems, we need an additional assumption concerning the sequence $\left\{\Delta x_{t}\right\}$.

A4:
$\sum_{i=1}^{N}\Delta x_{t}^{2}=\infty .$

Assumption A4 prevents situations in which the presence of too small increments of $x_{t}$ makes convergence of the algorithm too slow; in other words, we require that the signal is sufficiently exciting for the considered algorithm, see, e.g.,  [49]. Similarly, the assumption that $x_{t}$ is a random process leads to the condition $E\{\Delta x_{t}^{2}|{\hat{g}}_{t},{\hat{g}}_{t-1},...\}>0$, see [44,45].

**Remark** **11.** *Notice that the only formal restriction concerning the measured signal is given in A2 and A4. In principle, variability in the input signal may have positive effects on the calibration procedure (as in process identification; for example, see [49,61]).*

**Theorem** **3.** *Let the model (21) hold and let the assumptions A1–A4 be satisfied; let node 1 be a center node of the graph G. Then, the following are true:*

*(a) ∀t>0 $\exists \kappa_{1}^{\prime }>0$, such that $\forall (0<\kappa_{1}<\kappa_{1}^{\prime })$; the inequality $∥w_{t}^{-}∥<\kappa_{1}$ implies $\max_{l}R\left\{\lambda_{l}{\left(\Gamma_{t}^{w}\right)}^{-}\right\}<\kappa_{2}<0$, where $\lambda_{l}\left(\cdot \right)$ is the l-th eigenvalue of an indicated matrix.*

*(b) $\exists \kappa_{3}>0$, such that $\exists \delta^{\prime \prime }>0$, such that the model (21) is uniformly asymptotically stable $\forall (0<\delta <\delta^{\prime \prime })$, if, in addition, $∥\Delta x_{t+1}^{2}{\left(\alpha^{-}\right)}^{2}{\left(\Gamma_{t+1}^{w}\right)}^{-}-\Delta x_{t}^{2}{\left(\alpha^{-}\right)}^{2}{\left(\Gamma_{t}^{w}\right)}^{-}∥\;<\;\kappa_{3}$, ∀t>0.*

*(c) The model (21) is B.I.B.O.-stable.*

*(d) $∥s_{t}∥\;\to \;0$, when t→∞ and $∥w_{t}∥\;\to \;0$.*

**Proof.**  For (a) of the assertion, it is important to notice that the matrix ${\left(\alpha^{-}\right)}^{2}\Gamma^{-}$ is constant and Hurwitz (since $\max_{l}R\left\{\lambda_{l}\left({\left(\alpha^{-}\right)}^{2}\Gamma^{-}\right)\right\}<0$), e.g., [34,44,45]. Having in mind that the eigenvalues of a matrix are continuous functions of its elements, the eigenvalues of $\Delta x_{t}^{2}{\left(\alpha^{-}\right)}^{2}\Gamma_{t}^{w-}$ are continuous functions of the norm of $w_{t}^{-}$. Therefore, the assumption from Theorem 24.8 in [52] concerning the time-varying eigenvalues of the state matrices is satisfied, i.e., ∃μ<1, such that $|\lambda_{l}\left(\cdot \right)|<\mu ,\forall l,\forall t$.

**Remark** **12.** *The key assumption from Theorem 24.8 in [52] concerns the time-varying eigenvalues of the state matrices in linear time-varying state models (their module should be strictly less than one). Assertion (a) of the Theorem indicates how this general assumption can be applied to the time-varying dynamic model representing the algorithm.*

Assertion (b) follows from the Theorem 24.8 in [52]. Technically, one starts from the Lyapunov difference equation connected to (21) and shows, after standard technicalities, that the uniform asymptotic stability is achieved when the parameter variations are slow enough (according to the constant $\kappa_{3}$).

Assertion (c) follows from Theorem 27.6 in [52], stating that a linear dynamic input–output model is uniformly B.I.B.O.-stable if, and only if, it is uniformly exponentially stable.

Assertion (d) follows in a straightforward way.

Thus, the result follows. □

**Remark** **13.** *It is important to draw practical conclusions from the derived theoretical results. It is obvious that the gain calibration of a network based on the application of the full dynamic consensus scheme is not a reliable tool for blind sensor network calibration under nonlinear sensor characteristics—keeping in mind the fact that there is a high possibility of attaining divergence in the estimates. The introduction of a micro-calibrated sensor with a desired corrected gain represents a way of practically overcoming the stability problems and obtaining practically acceptable results at the expense of one additional micro-calibration. Furthermore, Theorem 3 shows that, when the algorithm step size and signal time variations are small enough, we can expect the stability of the algorithm and convergence to the neighborhood of the selected reference gain (see also Section 8).*

## 5. Simultaneous Calibration of Offsets and Gains

### 5.1. Consensus Algorithm

The previous sections have been devoted to offset and gain calibration taken separately, in order to gradually introduce the basic lines of reasoning. Following further the main ideas from the literature [34,35,36,44,45,46], it is possible to develop algorithms for the *simultaneous correction* of both offsets and gains in the presence of nonlinear sensor characteristics.

The calibration algorithms can be constructed in a straightforward way starting from Section 3 and Section 4. Namely, we define in the general case

(22) $${\hat{z}}_{t}^{i}={\hat{a}}_{t}^{i}y_{t}^{i}+{\hat{b}}_{t}^{i}={\hat{a}}_{t}^{i}\alpha^{i}x_{t}+v_{t}^{i}+{\hat{a}}_{t}^{i}\beta^{i}+{\hat{b}}_{t}^{i}$$

and $\varepsilon_{t}^{ij}={\hat{z}}_{t}^{j}-{\hat{z}}_{t}^{i}$, so that we have

(23) $$\varepsilon_{t}^{ij}=x_{t}\left({\hat{g}}_{t}^{j}-{\hat{g}}_{t}^{i}\right)+{\hat{f}}_{t}^{j}-{\hat{f}}_{t}^{i}+{\hat{g}}_{t}^{j}v_{t}^{j}-{\hat{g}}_{t}^{i}v_{t}^{i},$$

where ${\hat{g}}_{t}^{i}=\alpha^{i}{\hat{a}}_{t}^{i}$ and ${\hat{f}}_{t}^{i}={\hat{a}}_{t}^{i}\beta^{i}+{\hat{b}}_{t}^{i}$. It follows that, if the gain correction is performed by using the full-consensus algorithm from Section 4, we can aim to asymptotically attain ${\hat{g}}^{j}={\hat{g}}^{i}$ at consensus. This implies that ${\hat{f}}_{t}^{j}={\hat{f}}_{t}^{i}$, leading to the conclusion that the algorithm for simultaneous offset and gain correction is obtained by using both (4) and (16), modified in such a way that (4) includes an expression resulting from the gain-correction algorithm [44,45]. In other words, we now have the same calibration recursion as that shown in Section 4, in which we introduce ${\hat{z}}_{t}^{i}$, defined by (22), instead of ${\hat{z}}_{t}^{i}=y_{t}^{i}+\hat{b}$, adopted in Section 3. In terms of the corrected parameters, we have (17) for the gain parameters defined in Section 4 and the following complete recursion for ${\hat{f}}_{t}^{i}={\hat{a}}_{t}^{i}\beta^{i}+{\hat{b}}_{t}^{i}$, i.e.,

(24) $$\begin{array}{cc} {\hat{f}}_{t+1}^{i}= & {\hat{f}}_{t}^{i}+\delta \sum_{j}\gamma_{ij}\left({\hat{f}}_{t}^{j}-{\hat{f}}_{t}^{i}\right)+\delta x_{t}\sum_{j}\gamma_{ij}\left[{\hat{g}}_{t}^{j}\left(1+\frac{v_{t}^{j}}{x_{t}}\right)-{\hat{g}}_{t}^{i}\left(1+\frac{v_{t}^{i}}{x_{t}}\right)\right]\\ & +\delta \Delta x_{t}^{2}\alpha^{i}\beta^{i}\sum_{j}\gamma_{ij}\left[{\hat{g}}_{t}^{j}\left(1+w_{t}^{j}\right)-{\hat{g}}_{t}^{i}\left(1+w_{t}^{i}\right)\right]\left(1+w_{t}^{i}\right). \end{array}$$

At the sensor network level, we have the following vector–matrix model:

(25) $${\hat{f}}_{t+1}={\hat{f}}_{t}+\left(I+\delta \Gamma \right){\hat{f}}_{t}+\delta x_{t}\Gamma \left(I+{V\prime }_{t}\right){\hat{g}}_{t}+\delta x_{t}^{2}\alpha \beta \Gamma_{t}^{w}{\hat{g}}_{t}$$

where ${V\prime }_{t}=\mathrm{diag}\left\{\frac{v_{t}^{1}}{x_{t}},\ldots ,\frac{v_{t}^{n}}{x_{t}}\right\}$ and $\beta =\mathrm{diag}\{\beta^{1},\ldots ,\beta^{n}\}$.

The convergence properties of the recursions (17) and (25), based on full consensus, can be analyzed, directly extending the above-given results. The convergence of the first recursion has already been clarified. The dynamics of the second recursion follows from Theorem 1 after introducing a necessary modification, resulting from the fact that the offset estimate now contains an additional input term generated by (17). One easily concludes, therefore—on the basis of the results concerning the gain correction taken alone—that the composition of (17) and (25) is practically inapplicable.

### 5.2. Algorithm with Reference

Analogously with the preceding sections, it is possible to construct an algorithm for simultaneous offset and gain calibration based on the introduction of a reference node, now characterized by the pair $\left(f_{ref}^{1},g_{ref}^{1}\right)$. For gain calibration, we have again the recursion (21) for $s_{t}$; meanwhile, in the case of offset calibration, we have the following recursion derived from (24):

(26) $$\begin{array}{cc} r_{t+1}= & \left(I+\delta \Gamma^{-}\right)r_{t}+\delta x_{t}\Gamma^{-}\left(I+{V\prime }_{t}^{-}\right)s_{t}\\ & +\delta x_{t}^{2}\alpha^{-}\beta^{-}\left[{\left(\Gamma_{t}^{w}\right)}^{-}s_{t}+\Gamma^{-}\left(I+W_{t}^{-}\right)w_{t}^{-}\right], \end{array}$$

where $g_{ref}^{1}=1$ and $f_{ref}^{1}=0$, $\beta^{-}=\mathrm{diag}\left\{\beta^{2},\ldots ,\beta^{n}\right\}$ and ${V\prime }_{t}^{-}=\mathrm{diag}\left\{\frac{v_{t}^{2}}{x_{t}},\ldots ,\frac{v_{t}^{n}}{x_{t}}\right\}$.

The convergence properties of (26) depend on the convergence properties of (21).

**Theorem** **4.** *Let the assumptions of Theorems 2 and 3 hold. Then, $r_{t}$, generated by (26), is B.I.B.O.-stable; also, $∥r_{t}∥\;<\;\infty$ and $∥r_{t}∥\;\to \;0$, when t → ∞ and $∥v_{t}∥,∥w_{t}∥\;\to \;0$.*

The proof can be derived as a straightforward technical extension of the presented results.

## 6. Calibration in a Stochastic Environment Under Sensor Nonlinearities

The influence of nonlinear sensor characteristics in blind sensor network macro-calibration algorithms has been addressed above through a deterministic framework. In this section, we present the main lines of an extension of these results to the case of a stochastic environment; here, we assume two main stochastic components: (1) additive measurement noise superimposed on the sensor outputs; (2) additive communication noise superimposed on the signals sent between the network nodes ($z_{t}^{i}$ and/or $\Delta z_{t}^{i}$, i=1,…,n). We first present the possibility of a direct extension of the above-given results to the case of the existence of measurement and communication noises; then, the main characteristics of offset and gain calibration in the presence of noise without nonlinearities are presented, in order to emphasize their specific effects. We formally pay attention only to the case of the full-consensus algorithms, since these algorithms can be transformed into the algorithms with direct reference using the above-given methodology. We concisely present the basic theoretical conclusions here, and indicate their importance for practice, without going into all the details, as this would require much more space (see [60,61,62,63], together with [34,36,45,46,47]).

### 6.1. Assumptions and General Aspects

We adopt the following set of additional assumptions:

A5: Instead of $z_{t}^{j}$ and/or $\Delta z_{t}^{j}$ sent by the node *j*, node *i* receives the signal $z_{t}^{j}+\eta_{t}^{ij}$ and/or $\Delta z_{t}^{j}+\eta_{t}^{ij}$, where $\left\{\eta_{t}^{ij}\right\}$ are i.i.d. random sequences, independent of $x_{t}$ and measurement noise, satisfying $E\{\eta_{t}^{ij}\}=0$ and $E\left\{{\left(\eta_{t}^{ij}\right)}^{2}\right\}={\left(\sigma_{\eta }^{ij}\right)}^{2}<\infty$, i,j=1,…,n.

A6: Measurement noise sequences $\xi_{t}^{i}$ from (1) are mutually independent i.i.d. random sequences, independent of $x_{t}$ and communication noise, and satisfy $E\{\xi_{t}^{i}\}=0$ and $E\left\{{\left(\xi_{t}^{i}\right)}^{2}\right\}={\left(\sigma_{\xi }^{i}\right)}^{2}<\infty$, i=1,…,n.

A7: The step size sequences of the algorithms satisfy $\delta_{t}^{i}>0$, $\sum_{t=0}^{\infty }\delta_{t}^{i}=\infty$ and $\sum_{t=0}^{\infty }{\left(\delta_{t}^{i}\right)}^{2}<\infty$.

Assumption A7 is typical for stochastic approximation algorithms, enabling the successful asymptotic suppression of noise influence, e.g., [49,60].

In order to indicate a direct way of extending the results obtained in the preceding sections to the stochastic case, we have to recall that our calibration algorithms can be formally considered to be time-varying linear systems, containing the following: (1) stochastic inputs (measurement and communication noises); (2) deterministic inputs (nonlinearity terms). Responses to these inputs are to be superimposed onto each other.

The following lemma can be directly applied in an attempt to reconsider the above-presented results in a case when $\delta_{t}^{i}$ is not constant, but satisfies A7 instead of A1.

**Lemma** **3** ([64])**.** *Let a real number sequence, $\rho_{t}\geq 0$, satisfy the following inequality:*

(27) $$\rho_{t+1}\leq \left(1-\gamma_{t}\right)\rho_{t}+\gamma_{t}p_{t},$$

*where $0\leq \gamma_{t}\leq 1$ and $0\leq p_{t}\leq p$. Then,*

$$\rho_{t}\leq \rho_{0}\prod_{k=0}^{t-1}\left(1-\gamma_{k}\right)+p\left[1-\prod_{k=0}^{t-1}\left(1-\gamma_{k}\right)\right].$$

*If, in addition, ${lim sup}_{t\to \infty }p_{t}\leq p$ and $\sum_{t=0}^{\infty }\gamma_{t}=\infty$, then ${lim sup}_{t\to \infty }\rho_{t}\leq p$.*

Generalizations of Lemma 3 to time-varying vector–matrix recursions can be performed in a straightforward way, e.g., [50,51]. After additional technicalities, it is possible to prove that the above-derived results related to $\delta_{t}^{i}=\delta$ can be essentially extended to a case when $\delta_{t}^{i}$ satisfies A7, without any significant changes occurring in the already-adopted assumptions. Therefore, in the following text, we present the main results obtained through applying the analyzed calibration algorithms to linear sensor models in the presence of measurement and communication noise, assuming $v_{t}^{i}=0$ in (1).

### 6.2. Offset Calibration

**Theorem** **5.** *Let A2–A7 hold and let (1) hold with $v_{t}^{i}=0$, i=1,…,n, i.e., the model is linear and the measurement noise is present; let the communication noise be present, so that $z_{t}^{j}+\eta_{t}^{ij}$. Then, algorithm (5) converges to consensus in the mean square sense.*

**Proof.**  According to Section 3 and [33,34,36,45,47], now, we have for $\delta_{t}^{i}=\delta_{t}$, i=1,…,n the following vector–matrix recursion:

(28) $${\hat{f}}_{t+1}=\left(I+\delta_{t}\Gamma \right){\hat{f}}_{t}+\delta_{t}\zeta_{t},$$

where $\zeta_{t}=\mu_{t}+\nu_{t}$, with $\mu_{t}={\left[\mu_{t}^{1}\cdots \mu_{t}^{n}\right]}^{T}$, $\nu_{t}={\left[\nu_{t}^{1}\cdots \nu_{t}^{n}\right]}^{T}$, $\mu_{t}^{i}=\sum_{j\in N_{i}}\gamma_{ij}\left(\xi_{t}^{j}-\xi_{t}^{i}\right)$ (local measurement noise) and $\nu_{t}^{i}=\sum_{j\in N_{i}^{in}}\gamma_{ij}\eta_{ij}$ (local communication noise), i=1,…,n.

Let ${\tilde{f}}_{t}=T^{-1}{\hat{f}}_{t}$; similarly to Theorem 1, we obtain

(29) $$\begin{array}{ccc} & {\tilde{f}}_{t+1}^{\left[1\right]}={\tilde{f}}_{t}^{\left[1\right]}+\delta_{t}{\zeta \prime }_{t} & \\ & {\tilde{f}}_{t+1}^{\left[2\right]}=\left(I+\delta_{t}\Gamma^{*}\right)+\delta_{t}{\zeta \prime \prime }_{t}, \end{array}$$

where ${\zeta \prime }_{t}=\pi \zeta_{t}$ and ${\zeta \prime \prime }_{t}=S^{*}\zeta_{t}$, $\dim\{{\zeta \prime }_{t}\}=1$, $\dim\{{\zeta \prime \prime }_{t}\}=n-1$.

Define the Lyapunov functions $L_{t}^{\left[1\right]}=E\left\{{\tilde{f}}_{t}^{[1]2}\right\}$ and $L_{t}^{\left[2\right]}=E\left\{{\tilde{f}}_{t}^{[2]T}P{\tilde{f}}_{t}^{\left[2\right]}\right\}$, where P>0 is the solution to the algebraic Lyapunov equation, $P\Gamma^{*}+\Gamma^{*T}P=-Q^{*}$, for any given $Q^{*}>0$ (keeping in mind the fact that $\Gamma^{*}$ is, by assumption, a Hurwitz). It follows from A7 that

(30) $$L_{t+1}^{\left[1\right]}\leq L_{t}^{\left[1\right]}+C_{1}\delta_{t}^{2}\left(1+L_{t}^{\left[1\right]}\right),$$

(31) $$L_{t+1}^{\left[2\right]}\leq \left(1-\delta_{t}\lambda_{min}\left(Q^{*}\right)\right)L_{t}^{\left[2\right]}+C_{2}\delta_{t}^{2}$$

where $C_{1}>0$, $C_{2}>0$ and $\lambda_{min}\left(Q^{*}\right)>0$. It is possible to show that $L_{t}^{\left[1\right]}<\infty$ and $\lim_{t\to \infty }L_{t}^{\left[2\right]}=0$. Consequently, ${\tilde{f}}_{t}$ tends to a vector ${\left[{\tilde{f}}_{\infty }^{\left[1\right]}0\cdots 0\right]}^{T}$, where ${\tilde{f}}_{\infty }^{\left[1\right]}$ is a random variable satisfying $E\{{\tilde{f}}_{\infty }^{[1]2}\}<\infty$. Therefore, one concludes that ${\hat{f}}_{t}$ tends to $\chi_{\infty }1$ in the mean square sense, where $\chi_{\infty }$ is a scalar random variable satisfying $E\{\chi_{\infty }^{2}\}<\infty$. □

### 6.3. Gain Calibration

#### 6.3.1. Communication Noise

**Theorem** **6.** *Let A2–A7 hold and let (1) hold with $v_{t}^{i}=0$ and $\xi_{t}^{i}=0$, i=1,…,n; the communication noise is present, i.e., $\Delta z_{t}^{j}+\eta_{t}^{ij}$. Then, algorithm (17) converges to consensus in the mean square sense.*

**Proof.**  According to Section 4 and [33,34,36,45,47], we have now

(32) $${\hat{g}}_{t+1}=\left(I+\delta_{t}\Delta x_{t}^{2}\alpha^{2}\Gamma \right){\hat{f}}_{t}+\delta_{t}\Delta x_{t}^{2}\alpha^{2}\nu_{t}.$$

It is obvious from (32) that Theorem 5 can be directly applied after using the transformation $\tilde{T}$ (corresponding to matrix $\alpha^{2}\Gamma$), in the way transformation *T* is used in Theorem 5 in relation with matrix Γ. □

#### 6.3.2. Measurement Noise

In the case of gain calibration, measurement noise has a qualitatively different impact on the calibration results compared with the communication noise. Namely, the recursion for ${\hat{g}}_{t}$ now contains a term resulting from the multiplication of the identical noise terms in $\varepsilon_{t}^{\Delta ,ij}\Delta y_{t}^{i}$ and $\Delta y_{t}^{i}$, implying a nonzero parameter estimation bias and lack of convergence to consensus [33,34,36,45,47,49,61]. In order to overcome this problem, the *instrumental variable* methodology has been proposed, leading to the following alternative of (16):

(33) $${\hat{a}}_{t+1}^{i}={\hat{a}}_{t}^{i}+\delta_{t}^{i}\sum_{j\in N_{i}^{in}}\gamma_{ij}\varepsilon_{t}^{\Delta ,ij}Z_{t}^{i}$$

where $Z_{t}^{i}$ is the instrumental variable, which should be correlated with $\Delta x_{t}^{i}$ and uncorrelated with $\xi_{t}^{i}$ [49]. It has been shown that the choice $Z_{t}^{i}=\Delta y_{t-\tau }^{i}$ for τ≥2 is surprisingly simple and effective (see, e.g., the simulation results below). A compact form of the recursion for ${\hat{g}}_{t}$ is then

(34) $${\hat{g}}_{t+\tau }=\left(I+\delta_{t}\Delta x_{t}\Delta x_{t-\tau }\alpha^{2}\Gamma \right){\hat{g}}_{t}+\delta_{t}m_{t},$$

where τ≥2, $m_{t}={\left[m_{t}^{1}\cdots m_{t}^{n}\right]}^{T}$ with $m_{t}^{i}=\delta_{t}\sum_{j}\gamma_{ij}$$\left({\hat{a}}_{t}^{j}\Delta \xi_{t}^{j}-{\hat{a}}_{t}^{i}\Delta \xi_{t}^{i}\right)$$\left(\alpha^{i}\Delta x_{t-\tau }+\Delta \xi_{t-\tau }^{i}\right)$. It is not difficult to verify that $\left\{m_{t}\right\}$ is a zero-mean independent random sequence, as a consequence of the introduction of the instrumental variables. Consequently, we have the following important result:

**Theorem** **7.** *Let A2–A7 hold and let (1) hold with $v_{t}^{i}=0$, i=1,…,n; let the measurement and communication noises be present. Then, algorithm (34) converges to consensus in the mean square sense.*

The proof formally follows the proof of Theorem 5.

Assumptions for achieving B.I.B.O. stability do not impose any significant restriction in practice (pay attention to Assumptions A1–A3). Notice, however, that the B.I.B.O. stability does not presuppose, by definition, convergence to zero of the input variables. According to Assumption A2, we assume only the boundedness of the nonlinear term in a very general sense. We do not believe that, after proving the above general Theorems, any additional quantitative analysis of the error bounds has evident sense without specifying eventual specific aspects, including the characteristics of the sensor, the measured signal, and the environment.

## 7. Simulation Results

This section is devoted to the simulation results, illustrating the theoretical background presented in Section 2, Section 3, Section 4, Section 5 and Section 6. In this sense, the subsections given below correspond to the ordered sequence of the above-presented theoretical results. The main intention here is to illustrate the practical applicability of the analyzed distributed calibration algorithms under the presence of nonlinearities in sensor models. In order to emphasize the main effects, the adopted values of some variables and parameters are slightly out of the realistic range. The main focus here is to delineate, within all the presented types of algorithms, the distinction between full-consensus-based and reference-based recursions. The main practical idea has been to emphasize the important fact that only one additional local sensor micro-calibration allows us to obtain both theoretical stability and practical effectiveness.

All the results are obtained by simulating a ten-node network. In each experiment, a network digraph (together with its weights) is chosen at random from a large set of digraphs, satisfying Assumption A3. Sensor parameters $\alpha^{i}$ and $\beta^{i}$ have been selected at random to be about one and zero, respectively, with standard deviations equal to 0.1. The measured signal, $x_{t}$, has been generated by a simple second-order AR process, with a standard deviation of 1. As far as the nonlinearities are concerned, they should remain bounded according to A2. All the presented simulations are obtained by using nonlinearities composed of pieces of polynomials and exponential and trigonometric functions, chosen at random.

### 7.1. Offset Calibration

**Figure 1:**
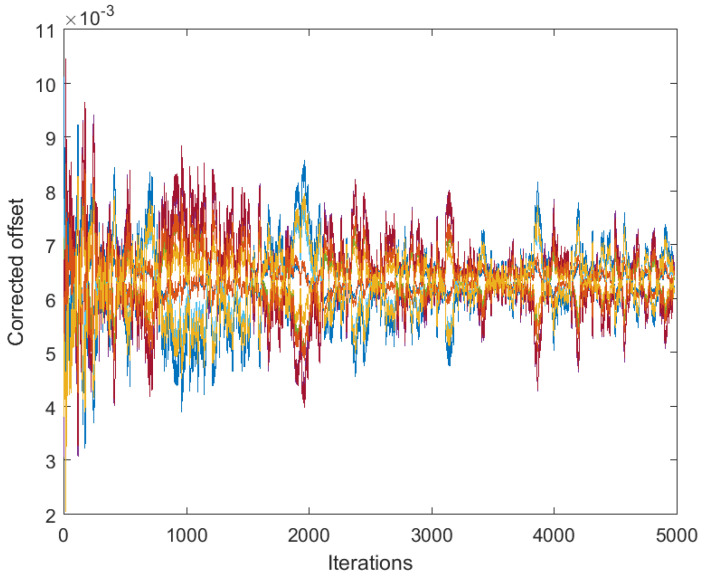
Time evolution of corrected offsets; full-consensus algorithm. Different colors represent different node’s estimates.

**Figure 2:**
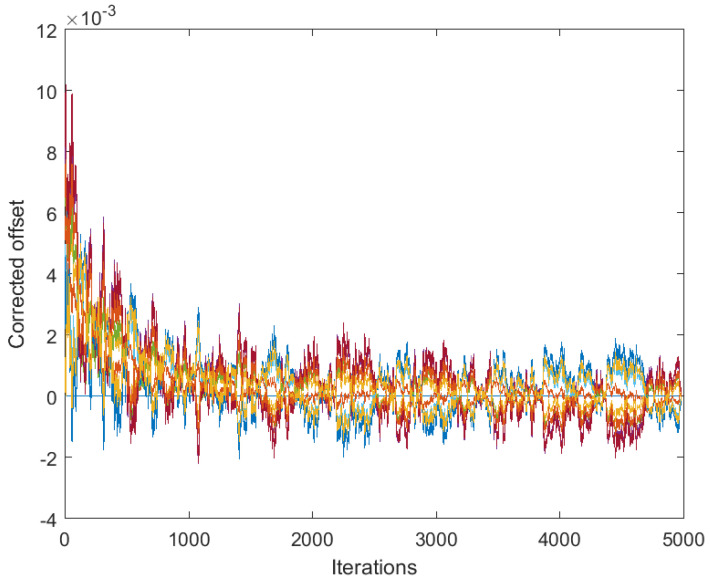
Time evolution of corrected offsets; algorithm with offset reference. Different colors represent different node’s estimates.

Figure 1 presents the results obtained by algorithm (4) based on the full consensus of the corrected offsets, and Figure 2 presents the results obtained by algorithm (10), (11) based on the introduction of one selected reference (node 1). The level of 0.15 was selected for $\max_{t}\left|v_{t}^{i}\right|$ (this is much higher than it would be in reality, in order to present the main effects more visibly), with a step size of δ=0.05. In both figures, the estimates behave in accordance with Theorems 1 and 2. Namely, in Figure 1, we can easily recognize the large bias identified in (7). On the other hand, Figure 2 shows the effect of selecting a reference offset that is equal to zero: all the parameter trajectories show an obvious convergence to zero. It is possible to note that the convergence rate is somewhat slower and the variance is higher in the case of Figure 2 (keeping a lower number of parameters in mind, i.e., a lower number of degrees of freedom in (11)).

### 7.2. Gain Calibration

**Figure 3:**
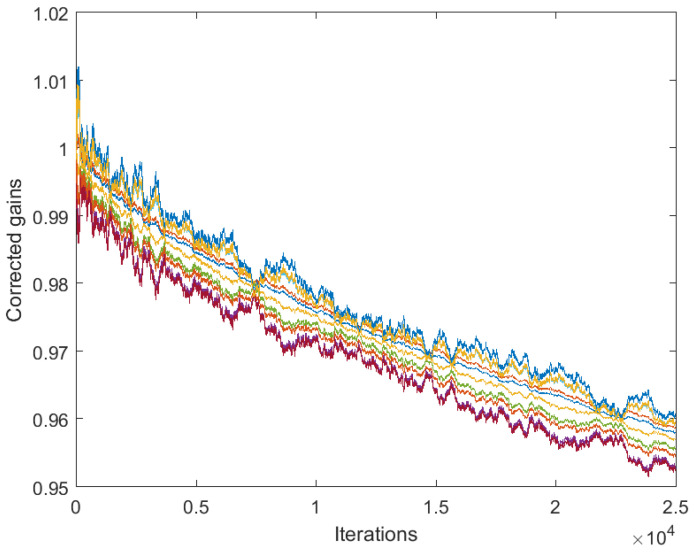
Time evolution of corrected gains; full-consensus algorithm. Different colors represent different node’s estimates.

**Figure 4:**
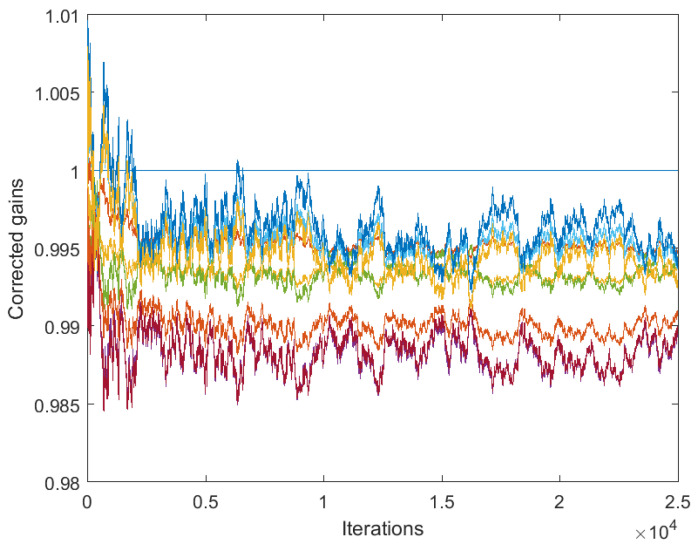
Time evolution of corrected gains; algorithm with gain reference. Different colors represent different node’s estimates.

Figure 3 and Figure 4 correspond to the algorithms for gain calibration, under all the assumptions adopted in Section 3.2, with the same level of the discrepancy between the linear model and the nonlinear one. Figure 3 illustrates divergence of the full-consensus-based algorithm (16), as theoretically indicated. Figure 4 illustrates how the introduction of a reference sensor tuned to $g_{ref}^{i}=1$ ameliorates calibration quality, showing practically acceptable behavior, in accordance with Theorem 3. It has been found by experimenting with different signals, $x_{t}$, that the requirement for slow time variations formulated in Theorem 3 does not impose any serious limitations in practice.

### 7.3. Simultaneous Offset and Gain Calibration

**Figure 5:**
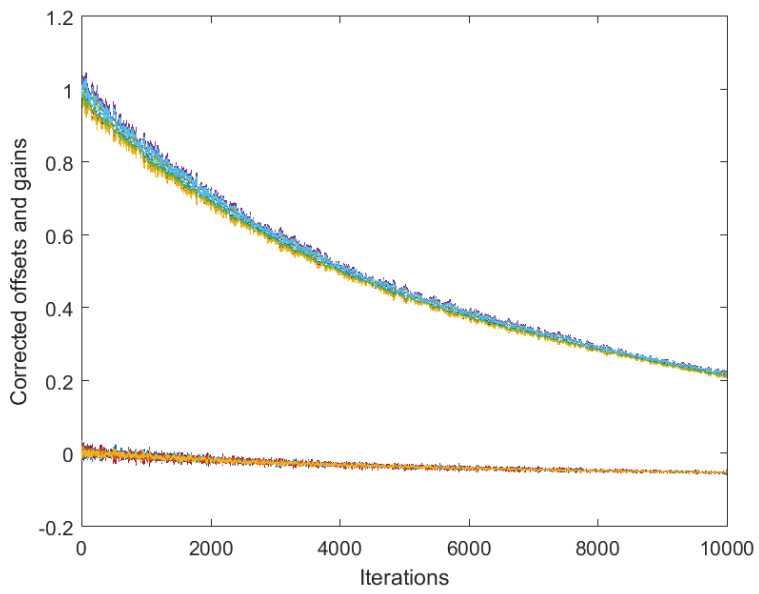
Time evolution of corrected offsets and gains; simultaneous offset and gain correction; full-consensus algorithm. Different colors represent different node’s estimates.

**Figure 6:**
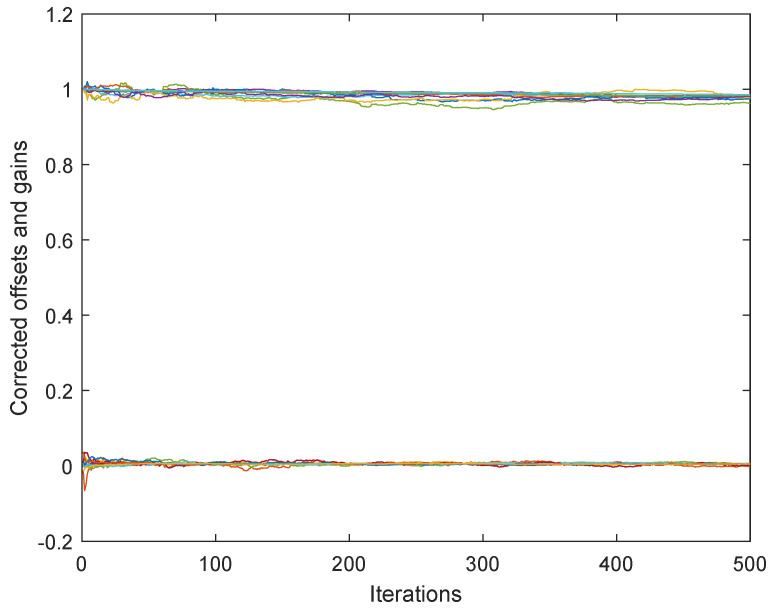
Time evolution of corrected offsets and gains; simultaneous offset and gain correction; algorithm with offset and gain references. Different colors represent different node’s estimates.

Figure 5 and Figure 6 correspond to the algorithm for simultaneous offset and gain calibration. Parameter trajectories in Figure 5 are obtained by algorithm (25) for offsets and algorithm (16) for gains. As explained above, algorithm (16) with full consensus shows fast divergence for gains, and somewhat slower divergence for offsets. The introduction of a micro-calibrated node as a reference for both offsets and gains shows stable results, close to the reference values. The latter case could be recommended for practice, with a low sensitivity to the influence of unknown sensor nonlinearities.

### 7.4. Simultaneous Offset and Gain Calibration with Measurement and Communication Noises

**Figure 7:**
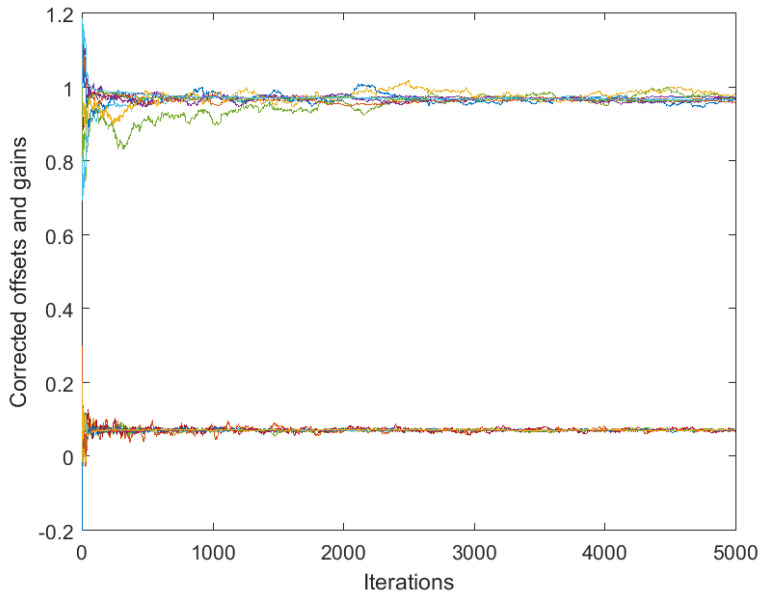
Time evolution of corrected offsets and gains; simultaneous offset and gain correction; measurement and communication noise; full-consensus algorithm. Different colors represent different node’s estimates.

**Figure 8:**
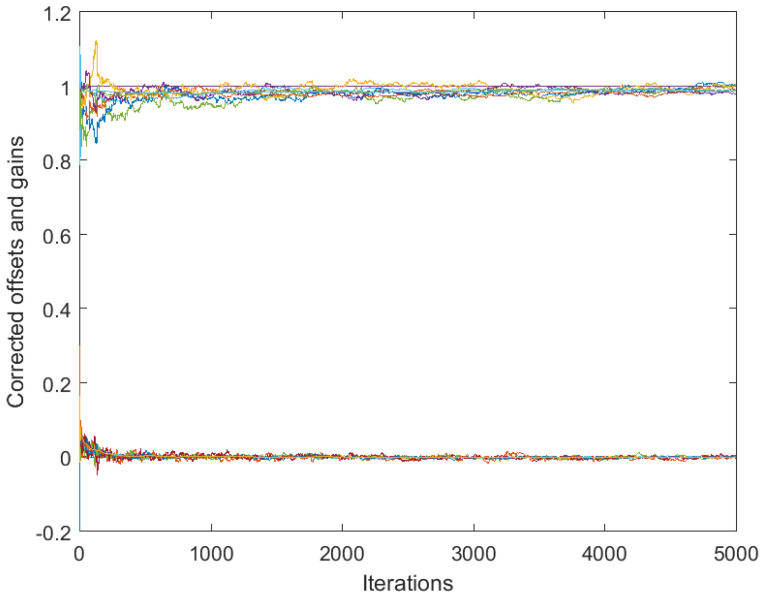
Time evolution of corrected offsets and gains; simultaneous offset and gain correction; measurement and communication noise; algorithm with offset and gain references. Different colors represent different node’s estimates.

Figure 7 and Figure 8 correspond to the case of the existence of measurement and communication noises when the offsets and gains are calibrated simultaneously in the absence of sensor nonlinearities, in order to emphasize the impact of noise alone. Both types of noise with a variance of 0.1 have been generated. In the case of gain calibration, the algorithm with instrumental variables has been applied with τ=2, using (34); the recursion (4) has been applied to offset calibration. In both cases, the step size has been defined as $\delta_{t}=1/\left(\mu +\nu t^{q}\right)$, μ=1, ν=1, q=0.6, in order to suppress noise influence and, at the same time, to achieve a satisfactory convergence rate. Obviously, in the case of full consensus, we have an evident divergence of the estimates; meanwhile, in the case of the algorithm with the reference, we can observe stable behavior, with the error depending on the noise level. For higher values of *q* (close to one), one can obtain much smoother curves, but these come at the expense of a lower convergence rate.

## 8. Calibration of Instrumentation for Aerodynamic Testing

In this section, we give a description of one specific application of sensor networks aiming to achieve pressure sensing in the aerodynamic testing of various objects. A complex multichannel high-performance pressure-measuring instrument has been developed within Project *MEMS, Multisensor Instrument for Aerodynamic Pressure Measurements*. The application of this instrument in practice is very specific, not allowing simple re-calibration in normal operating conditions. Moreover, issues of generating or measuring reference signals for standard calibration in real time remain questionable. We have found that the application of the blind calibration algorithms presented and analyzed in this paper could be very efficient in the case of the developed multichannel instruments. We present initial, encouraging results, showing that our calibration methodology could provide a good tool for distributed aerodynamic pressure sensing in normal operations.

### 8.1. Multichannel Aerodynamic Pressure-Sensing Instrument: General Description

*Aerodynamic testing* of fast-moving objects, such as aircraft and road or railway vehicles, is a necessary step in their development [65,66]. Some large stationary objects, such as civil engineering and architectural structures (bridges, buildings etc.), are also subjected to aerodynamic tests in order to estimate the influence of wind on their stability [67]. The tests can be performed either in a *wind tunnel* or in open space. A wind tunnel is a facility that generates a controlled air flow around the object under test, so as to produce aerodynamic effects that approximate those occurring in a real environment. Some wind tunnels are big enough to hold full-sized vehicles or other large objects, while many of them can only be used for testing of scaled models. Wind tunnels provide a well-controlled environment in terms of air flow quality, and can offer a wide flow velocity range, which would be difficult to achieve in open space.

*Multichannel pressure-measuring instruments* are essential for performing aerodynamic tests. In general, a multitude of small openings (pressure taps) are made on the aerodynamic surfaces of the tested object, and the taps are connected via pneumatic tubing to the instrument that measures the corresponding pressure values and sends the measurement signals to an acquisition system. Such instruments are usually called pressure scanners, because their early implementations had only one pressure sensor and an electromechanical valve for multiplexing of pressure channels, so that the channels were scanned, i.e., measured, one by one in a rapid succession. Contemporary pressure scanners are typically based on miniature MEMS pressure sensors, and use a separate sensor for each pressure channel. In terms of sensor signal processing, most instrument implementations perform only analog multiplexing and conditioning (typically amplification). In that case, the acquisition system receives the multiplexed analog signal, digitizes it, and stores the measured values. More demanding operations, such as sensor linearization, are performed in the digital domain on the stored measurement data. Pressure scanners of the latest generation digitize the measurement signals, perform digital signal processing internally, and send measurement data to an external computing equipment via a high-speed digital interface.

**Figure 9:**
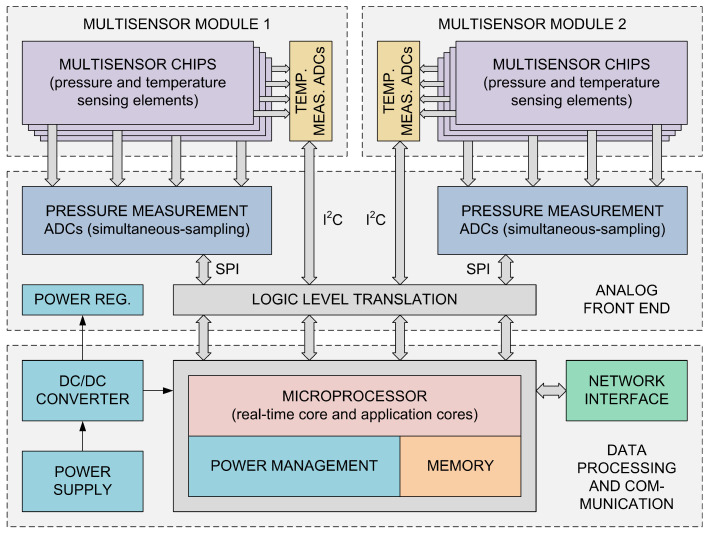
Simplified block diagram of the novel multichannel aerodynamic pressure scanner.

**Figure 10:**
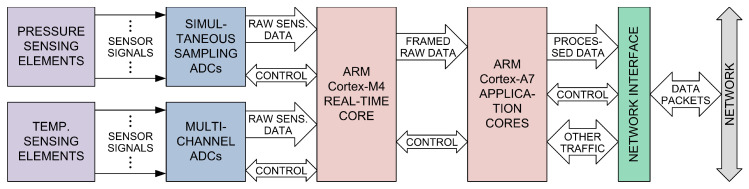
The flow of sensor signals and data in the instrument.

Increasing requirements, especially regarding the miniaturization of the instrument (the highest possible number of pressure measurement channels per unit volume, as well as the instrument’s overall dimensions), scanning speed (the number of samples per channel per second), and low measurement uncertainty, are difficult to meet at the same time, relying only on existing concepts and solutions. This fact has motivated researchers at the Department for Microelectronic Technologies (Institute of Chemistry, Technology and Metallurgy, University of Belgrade) to develop a novel pressure scanner concept, based on MEMS multisensor chips [68], simultaneous measurement at all pressure channels, and advanced digital signal processing. A block diagram of the novel pressure scanner designed according to this concept is shown in Figure 9. Two exchangeable multisensor modules contain several MEMS multisensor chips. Each chip has an array of piezoresistive MEMS pressure-sensing elements, as well as one or more resistive temperature-sensing elements; all of these are monolithically integrated on the same silicon die. Signals from the temperature-sensing elements are digitized by circuitry built into the multisensor modules; meanwhile, the conditioning and analog-to-digital conversion of signals from the pressure-sensing elements are performed by the analog front-end module. The latter consists of multichannel simultaneous-sampling 24-bit analog-to-digital converters, sensor excitation sources, and accompanying circuitry. The signals from all the pressure-measurement channels are sampled simultaneously, and the readout of the measurement data from the analog-to-digital converters is carried out by the data processing and communication module, where digital signal processing is performed in real time. Simultaneous measurement on all pressure channels is an important advantage of the instrument’s architecture for two reasons: (1) it eliminates the multiplexing of sensor signals, and thus its inherent disadvantages (parasitic effects, limited scanning speed); (2) it enables the performance of spatially coherent measurements in aerodynamic experiments that involve unsteady flow fields and transient phenomena. The data processing and communication module is based on a highly integrated system-in-package (OSD32MP157, Octavo Systems LLC, Houston, Texas, USA), which contains a microprocessor (two ARM Cortex-A7 application cores and one ARM Cortex-M4 real-time core), RAM, and power management circuitry. This system-in-package occupies a significantly smaller surface area compared to functionally equivalent solutions based on separate components. It was chosen in order to minimize the dimensions of the data processing and communication module, thus contributing to the instrument’s miniaturization. The Cortex-A7 application cores perform digital signal processing of the measurement data by applying sensor-correction algorithms based on the parametric models of sensing elements and the algorithms presented in this work. With operating frequencies of up to 800 MHz, these processor cores have high computing power, and thus do not pose any limit to the achievable measurement throughput of the instrument. The data processing and communication module communicates with external computing equipment, and transfers the processed measurement data to it via the computer network. This module also contains all the necessary power supply and interface circuitry. A diagram showing the flow of sensor signals and data in the instrument is given in Figure 10.

### 8.2. Instrument Calibration Using the Analyzed Algorithms

Semiconductor-based piezoresistive MEMS pressure sensors exhibit inherent temperature dependence [69], requiring suitable temperature compensation in practice. Similarly to other types of sensors, there are also deviations from the ideal linear model, including offset, $\beta^{i}$, gain, $\alpha^{i}$, and nonlinearity, $v_{t}^{i}$, which must also be minimized. The best results can be achieved by using digital signal-processing methods. In the case of the above-mentioned pressure scanner, we use a method based on a polynomial model, whose parameters are determined by *initial calibration*. In general, the initial calibration is performed by measuring the output signal values of all individual pressure-sensing elements at a multitude of different pressure and temperature values, distributed over the respective operating ranges of the instrument. The recorded values are then used to determine the mathematical model parameters for each of the sensing elements. Depending on the choice of model, this method minimizes the offset, the temperature drift of the offset and gain, and the sensor nonlinearity, resulting in measurement uncertainties lower than 0.1 % in a wide temperature range (see (1)).

However, being connected to a fixed moment of time, the initial calibration cannot take into account internal or external time-varying changes of the instrument characteristics, which can lead to small drifts in offset and/or gain. In the most demanding applications, the influence of these drifts on the measurement performance can be significant, so that *re-calibration* (after the initial calibration) has to be performed during operation. However, re-calibration in the classical sense, i.e., performed by applying a set of known pressure values, is not feasible during typical aerodynamic experiments, so that there is a need for a different approach, i.e., for blind calibration. The algorithms presented in this work are well suited for this purpose by the very definition of the adopted design methodology. There are two important additional aspects connected to the above-presented pressure-sensing instrument:

(1) The instrument utilizes multisensor chips with several pressure-sensing elements instead of a general array of discrete pressure sensors; thus, it follows that there is an *inherent similarity between the monolithically integrated pressure-sensing elements* in terms of their characteristics, i.e., formally, the parameters $\beta^{i}$, $\alpha^{i}$, and nonlinear terms $v_{t}^{i}$ in the sensor models (1) can be expected to be very close to each other for all *i*. It follows then, in this case, that Remarks 5 and 10 (given above) can be applied, so that one can achieve a significant reduction in the influence of the nonlinearities to the entire calibration procedure.

(2) Aerodynamic testing experiments are usually performed in such a way that there are time intervals, $T_{k}\geq 10\;\min$, k=1,2,… (typically after each consecutive measurement is taken), during which all the sensing elements are exposed to the *same slowly time-varying pressure signal*, $x_{t}$. Consequently, the general real-time calibration strategy for the described instrument can be based on applying the developed algorithms regularly during each $T_{k}$, k=1,2,…, eliminating any need for considering the difficult problem of constructing a spacial model for the signals in the general case (which itself requires complex offline computation).

A static nonlinearity is included in a very general way in the parametric mathematical model of the pressure sensors, according to (1). Such a definition encompasses a very large range of nonlinear functions, under the only assumption that the nonlinear term is bounded. This nonlinearity is always reduced through the initial calibration (the measurement uncertainty is less than 0.1% before the application of the described blind calibration algorithms).

In the case of silicon piezoresistive MEMS pressure sensors operating in air, i.e., without a separation membrane and oil filling (the type used in the described instrument), hysteresis is negligible, because single-crystal silicon can be considered to be an ideally elastic material, up to the breaking point (the rupture of the sensor’s diaphragm.) Therefore, the consideration of hysteresis and other memory effects in sensors is not needed in this case.

As far as the piece-wise nonlinearities are concerned, they belong to the bounded nonlinearities, provided that A2 is satisfied. All the above-presented simulations are obtained by using nonlinearities composed of pieces of polynomials and exponential and trigonometric functions chosen at random (see Section 7).

**Figure 11:**
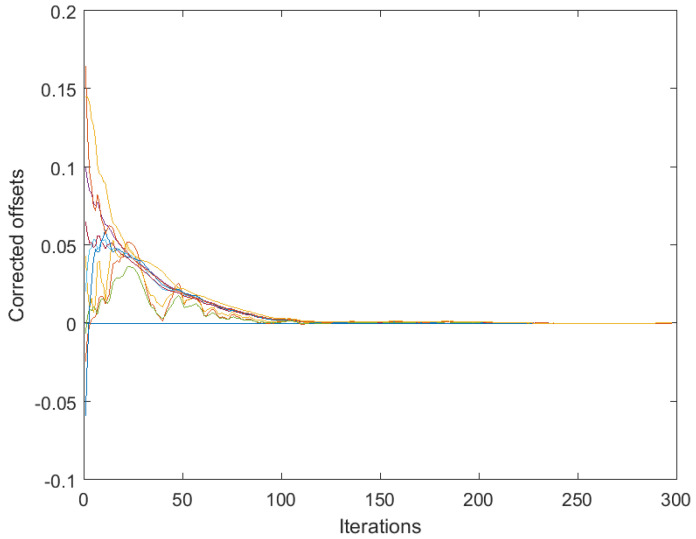
Time evolution of corrected offsets; algorithm with reference; multichannel pressure scanner. Different colors represent different node’s estimates.

According to practice, the online real-time calibration of the instruments like the presented one is primarily concerned with offset corrections; gain corrections are less frequently needed. Keeping this in mind, we performed extensive simulations and experimenting in order to test the calibration algorithms presented in Section 3 under circumstances close to reality, encompassing the nonlinearity terms, $v_{t}^{i}$, and measurement noises, $\xi^{i}$; thus, we assumed that the communication noise can been neglected (since communications between sensors are mostly performed internally within the multichannel instruments themselves). Figure 11 presents the time evolution of the corrected offsets generated by the algorithm with reference $f_{ref}^{1}=0$, implemented according to (4). The following conditions have been adopted: $∥v_{t}^{i}∥+∥\xi_{t}^{i}∥<0.1\%$ of the full-scale approach (as mentioned above); $\delta_{t}=1/\left(\mu +\nu t^{0.55}\right)$ with μ and ν found by trial and error; sampling rate equal to 10 Hz; $\gamma_{ij}$ chosen in such a way as to provide equal weights to all the nodes; network topology is chosen at random, according to A3. It is possible to note from the figure that the convergence to the predefined reference values is fast and accurate, with a very low variance.

**Figure 12:**
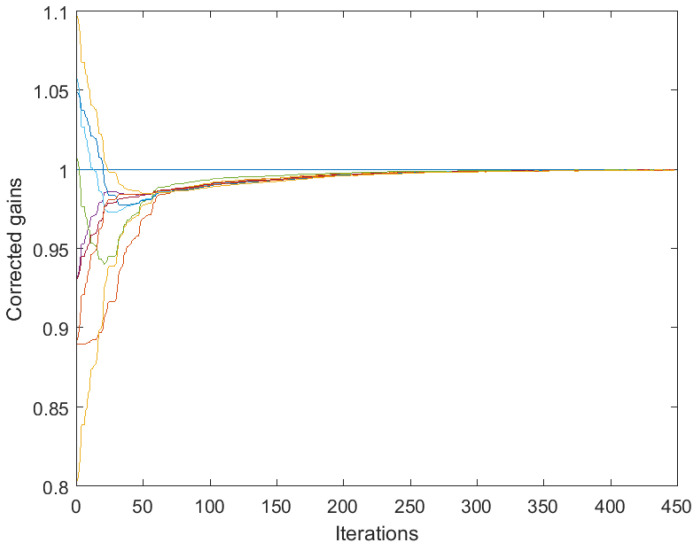
Time evolution of corrected gains; algorithm with reference and instrumental variables; multichannel pressure scanner. Different colors represent different node’s estimates.

In addition, Figure 12 gives the evolution of the corrected gains obtained by the algorithm with reference presented in Section 4. It has been adopted that $g_{ref}^{1}=1$, while the algorithm is implemented according to (16), with instrumental variables and τ=2. Convergence to the desired value is obvious.

It must be emphasized that the computational effort required by the proposed calibration algorithm is minimal. In order to shed full light on this important aspect, we present in the following text the pseudo-code (Algorithm 1). for all the computations performed in one iteration of the algorithm. The hardware in which the algorithms are embedded is more than sufficient to meet the needs of the proposed algorithms.

Both examples indicate that the consensus-based calibration methodology, using selected reference values, can be successfully applied to the developed multichannel instrument as a reliable real-time calibration tool.
**Algorithm 1** Computation of the calibration parameters at node *i*.
Initial values ${\hat{a}}_{0}^{i},{\hat{b}}_{0}^{i},Z_{0}^{i}$, step sizes $\delta_{t}^{i},t>0$, weights $\gamma_{ij}$, τ=2 Initialize step counter *t*
Repeat For all i∈{1,…,n} do Observe measurements $y_{t}^{i}$ and $\Delta y_{t}^{i}$ Compute $z_{t}^{i}$ and $\Delta z_{t}^{i}$ Send $z_{t}^{i}$ and $\Delta z_{t}^{i}$ to all out-neighboring nodes Obtain data $z_{t}^{j}$ and $\Delta z_{t}^{j}$ from all $j\in N_{i}^{in}$ Update ${\hat{a}}_{t+\tau }^{i}\;\leftarrow \;$${\hat{a}}_{t}^{i}+\delta_{t}^{i}\sum_{j\in N_{i}^{in}}\gamma_{ij}\varepsilon_{t}^{\Delta ,ij}Z_{t}^{i}$ according to (33) Update ${\hat{b}}_{t+1}^{i}\;\leftarrow \;$${\hat{b}}_{t}^{i}+\delta_{t}^{i}\sum_{j\in N_{i}^{in}}\gamma_{ij}\varepsilon_{t}^{ij}$ according to (4) End for Update the iteration counter t←t+1 Until convergence


### 8.3. Scalability

The scalability issue is, in principle, very important for practice. This issue has been elaborated in extenso in a number of previously published papers, e.g., [33,34,36]. It has been found in the literature that blind calibration algorithms that are compatible with the ones analyzed in this paper show a linear increase in the number of required operations, with the number of nodes, *N*, provided the sizes of in- and out-neighborhoods of all the nodes remain bounded. Formally speaking, it appears that the network topology is irrelevant for the convergence points of the algorithms (in the sense of the given theorems), as long as the graphs satisfy Assumption A3. However, the graph connectedness influences the convergence rate to consensus, which is very important in practice. Obviously, an increase in the connectedness degree of the graph leads to an increase in the convergence rate. However, in the case of typical applications of the designed instrument for pressure sensing, we do not have problems of this kind, since we do not expect more than 10 complete instruments to be present, even in the most complex experiments we can envisage (one instrument can contain more sensors, depending on the end users’ specifications). In practice, scalability is a more important and acute issue in large sensor networks dedicated to different large space monitoring tasks.

**Remark** **14.** *Sudden changes in the input signal may exist in aerodynamic pressure-sensing applications. This aspect is adequately covered by the instrument’s design, i.e., by choosing a sufficiently high sampling rate, and by using the type of sensors capable of withstanding high over-pressure values. However, the presented algorithms deal with offset and gain variations that are—by several orders of magnitude—slower than the input signal.*

## 9. Conclusions

The main original contributions presented in this paper can be summarized as follows:

- This paper achieved the incorporation of a static nonlinearity in the sensor models utilized in the design of a class of distributed consensus-based blind macro-calibration algorithms for sensor networks, initially formulated in [33,34,35,36,37,43,44,45,46,47].
- This paper presents a rigorous theoretical analysis of the algorithm for offset calibration belonging to the given class of sensor nonlinearities, including a proof of stability in the B.I.B.O. sense; spacial attention is paid to: (a) the calibration algorithm based on full consensus; (b) the calibration algorithm based on a predefined micro-calibrated reference node.
- This paper achieves the derivation of the explicit formulae for corrected offsets as functions of time.
- This paper presents a rigorous theoretical analysis of the gain calibration algorithm belonging to the given class under the presence of sensor nonlinearities, including: (a) a demonstration of the instability of the gain calibration algorithm based on full consensus; (b) a proof of its B.I.B.O. stability of the calibration algorithm based on a gain reference.
- This paper demonstrates the formulation of an algorithm for the simultaneous calibration of offsets and gains, together with a proof of the B.I.B.O. stability for a case of an algorithm with references for both gain and offset.
- This paper proposes a general methodology for incorporating stochastic disturbances in sensor models together with sensor nonlinearities, including the formulation of an appropriate lemma, opening up new methodological possibilities for extending the obtained results.
- This paper provides comprehensive illustrative simulation analyses of all the theoretically derived conclusions.
- This paper presents a newly designed multichannel aerodynamic pressure-sensing instrument.
- This paper presents the functioning of the newly developed instrument under normal operating conditions.
- This paper demonstrates that the proposed algorithms can be a basis for distributed online real-time blind re-calibration of large sensor networks under normal operating conditions, applicable to complex experiments with the developed pressure-sensing instrument.

## Data Availability

Data are contained within the article.
